# Patient’s experiences of diabetes care at a tertiary health facility in Lilongwe, Malawi

**DOI:** 10.1186/s12913-023-10039-z

**Published:** 2023-10-12

**Authors:** Ellen Nkambule, Kondwani Wella, Annie Msosa, Balwani Chingatichifwe Mbakaya, Evelyn Chilemba, Gladys Msiska

**Affiliations:** 1 St John’s Institute for Health, P.O. Box 18, Mzuzu, Malawi; 2 Kamuzu University of Health Sciences, P/Bag 1, Lilongwe, Malawi; 3 Department of Public Health, University of Livingstonia, Mzuzu, Malawi

**Keywords:** Diabetes, Insulin, Rural areas, Diabetes care, Narrative inquiry, Malawi

## Abstract

**Background:**

Little is known about experiences of rural people with diabetes care at a tertiary health facility in low-income settings. Understanding their experiences is essential for developing effective diabetes care interventions.

**Methods:**

The study employed a qualitative narrative inquiry. Participants were identified at a diabetes clinic at a tertiary-level healthcare facility. Ten participants from the rural areas attending the diabetes clinic were purposively selected. Data were collected through in-depth interviews in the privacy of the homes of the study participants and analysis was done using the Riessman approach to thematic narrative analysis.

**Results:**

In this study, the following four themes emerged: (1) the long pathway to a diagnosis of diabetes; (2) Poverty-related hardships and diabetic clinic attendance; (3) The impact of health worker attitudes and behavior on diabetes care; and (4) Low resources and their impact on self-management.

**Conclusions:**

Rural-based patients living with diabetes encounter enormous challenges as they access diabetes care. One of the challenges is delayed diagnosis of diabetes. There is a need to introduce point-of-care (POC) testing to improve diabetes diagnosis. In addition, there is a need to strengthen awareness campaigns among the population so that people are well informed about the signs and symptoms of diabetes to promote early diagnosis. Diabetes care must be decentralized from tertiary hospitals to primary health centers. This will improve access to diabetes care and reduce the burden associated with traveling a long distance to access diabetes care in Malawi.

**Supplementary Information:**

The online version contains supplementary material available at 10.1186/s12913-023-10039-z.

## Background

In sub-Saharan African countries, non-communicable diseases (NCDs) such as diabetes and hypertension have become an increasing public health concern and are expected to surpass HIV/AIDS as the leading cause of death in 2030 [1]. There is evidence of a growing burden of diabetes in Malawi. Studies conducted in the 1960 and 1970 s demonstrated that diabetes was an important health problem with an estimated prevalence of 1% or less [2]. The prevalence in sub-Saharan Africa (SSA) in 2015 was 6% [3]. However, in 2017 it was estimated to be 5.6% [4] and was slightly higher in rural than urban areas among adults aged between 25 and 64 years; 5.4% versus 4.4%, respectively [4].

Malawi is classified as one of the least-developed countries in the world, with 85% of its population living in rural areas [5]. It is indicated that 71% of Malawi’s 17 million people live in extreme poverty and most of them are concentrated in rural areas where there are limited economic activities ( [5] and where they reside far from secondary and tertiary healthcare facilities which provide specialist care. Consequently, rural-based patients living with diabetes have insufficient access to care and there is evidence to that effect [6]. Additionally, there is delayed diagnosis and the rural-based patients receive substandard diabetes care compared to their urban counterparts [7]. Evidence suggests that narratives about clinical encounters give opportunities for health professionals to listen to patients’ experiences of care and make necessary improvements [8]. However, there is a lack of in-depth knowledge about the experiences of diabetes care among rural-based diabetic patients at a tertiary facility in Malawi. The available literature on diabetes care focused on the assessment of quality of diabetes care at a secondary level health facility in Malawi [9]. This study, therefore reports on the experiences of rural-based patients living with diabetes on diabetes care at a tertiary health facility in Lilongwe, Malawi.

## Methods

### Research approach and design

This study adopted a qualitative narrative inquiry approach. In this approach, researchers describe the lives of individuals, collect and tell stories about people’s lives, and write narratives of individual experiences [10, 11]. This research approach was chosen because it is participant-centered. As argued by Haydon [12], there is a need to find what is important from the patients ‘point of view’ to optimize care. Polit and Beck [13] add that the broad underlying premise of narrative research is that people most effectively make sense of their world and communicate these meanings by constructing, reconstructing, and narrating stories. Furthermore, humans naturally communicate and express the depths of their identities, relationships, emotions, and beliefs through the telling of stories [13]. Thus, narrative inquiry provided the opportunity for participants to share what was important to their situation.

### Study setting

Participants were recruited at a diabetes clinic at a tertiary-level healthcare facility in Malawi. Tertiary facilities provide specialist health services at the regional level and also offer referral services to district hospitals within the region [14]. Participants from various rural areas in Lilongwe attend the diabetic clinic at Kamuzu Central Hospital (KCH). KCH diabetic clinics are scheduled two times a week (Tuesdays and Fridays), with an average of 40 to 60 patients living with diabetes per clinic session. Nurses and clinicians review these patients. We followed the participants to their respective homes for data collection. As observed by Polit and Beck [15], interviewing participants in their homes helps to avoid manipulation of the participants as they will be free to express themselves within their home setting. Interviewing participants at their residences was also consistent with the narrative inquiry research design.

### Sampling

The study population comprised adult patients living with diabetes from rural areas in Lilongwe. The inclusion criteria were (1) patients living with diabetes managed on insulin therapy (2) aged 18 years and older; (3) living with diabetes managed on insulin for more than 6 months; and (4) living in rural areas. We used purposive sampling. The sample consisted of 10 participants. Moule and Goodman [16] support a smaller sample size of 6–10 in a qualitative study for rich experiences and Creswell [17] recommends 1 to 2 individuals for narrative inquiry. Data saturation was achieved with a sample of eight because the participants were good informants who were able to share their lived experiences and communicated effectively. Two more participants were interviewed just to further verify data saturation. As noted by LoBiondo-Wood and Huber [18], it was known that saturation had been reached when the ideas surfacing in the dialogue were similar to those previously heard from other participants.

### Data collection instrument

Data were collected using a semi-structured interview guide. The semi-structured interview guide allowed the researcher to prepare a written topic guide, which is a list of areas or questions to be covered with each participant [15]. The questions focused on the following: experiences of living with diabetes managed on insulin therapy in rural areas; patients’ living with diabetes perceptions of their illness experience in rural areas; factors that enable and hinder coping with the illness in rural areas. Probes were asked when necessary. The semi-structured interview guide was developed in English and translated to Chichewa.

### Pretesting of the data collection tool

The interview guide was pretested with three rural-based adult patients living with diabetes managed on insulin therapy. Participants were identified at Kamuzu Central Hospital diabetes clinic and data were collected in their respective homes. Polit and Beck [15] stipulate that the pretesting of the interview guide helps to check the structure and order of the questions. Pretesting also helped the researcher to gain some practice in interviewing participants [19]. In this study, data from pretesting the interview guide were used with the rest of the data from the main study. As stated by van Teijlingen and Hundley [20] data from the pretesting studies are not used in the main studies because of contamination. Nevertheless, van Teijlingen and Hundley add that contamination is not a big concern in qualitative studies; and data from the pretest studies can be used in the main studies. Therefore, the researcher decided to include pretest interview data in the analysis with the rest of the data.

### Ethical considerations

The research was approved by the University of Malawi College of Medicine Research and Ethics Committee reference number P.03/20/2969. Further ethical approval was obtained from the study setting, tertiary hospital. Before participation, verbal and written informed consent was sought from the participants. To ensure anonymity, participants were assigned codes and names were not used in the study. After data analysis, the transcripts were kept according to the university’s data management procedures.

The Word Health Organization does not encourage incentives beyond reimbursements for expenses incurred as a result of participation in the research. These may include travel costs and reimbursement for time lost. In this study, data were collected in the privacy of the homes of the study participants. They were given refreshments and snacks as compensation for their time.

### Recruitment process

Recruitment of participants started after obtaining ethical approval from COMREC. The researcher submitted a copy of the ethical approval to the Hospital Director from whom permission to conduct the study was sought prior to submission of the research protocol to COMREC. A copy of the ethical approval and the letter of permission from the Hospital Director were shared with the Unit Chief Nursing Officer and the Unit In-charge of the Medical Clinic. Participants were identified at the medical diabetic clinics when they were attending the diabetic clinic. The researcher approached the prospective participants at the clinic in liaison with the Unit in charge of medical clinic and asked the patients to participate in the study. The researcher had a private meeting with the patients who accepted to participate in the study. During the meeting, the researcher explained the aims of the study, type of data, procedures and why the participant was chosen to be the prospective participants for the study. The investigator reviewed patient’s health passports to confirm their eligibility for the study. When the participants met the inclusion criteria, the investigator informed participants that the hospital was an access point but the interviews were to take place in their homes for which consent was sought. The researcher made necessary arrangements including accompanying the patient to their home to know the place. The investigator arranged for a date and time that was suitable for the participant for the interview. No participant declined to participate in the interviews.

### Data collection

Data were collected through in-depth face-to-face interviews by E.N. E.N has experience with qualitative research. Data were collected in the privacy of the homes of the rural-based diabetic patients. The interviews were conducted in Chichewa, a local language used in the area where this study was conducted. The description of the interview meeting with the participants was as follows: upon entering the house of the participant, the researcher spent the first three minutes discussing casual issues such as the status of the coronavirus pandemic and farming. This facilitated establishing a sense of rapport. The interviews commenced by confirming to each participant that he/she was ready to be interviewed. The next step was to explain the details of the study to the participant. The researcher read participants’ information sheets for them. Thereafter, the participants were asked if there were issues to be clarified or if they had any questions regarding the study. The researcher answered all the questions the participants had asked and then verbal and written consent was obtained. At the beginning of the interview, the researcher showed the participants the tape recorder, which was used to record the discussion, and informed the participant how the tape recorder works. The interviewer sought consent from participants for the use of a tape recorder. Generally, the researcher started by obtaining the demographic characteristics of respondents. Then the researcher introduced questions from the interview guide and used probing questions to seek clarifications and open up the interview. Although some participants were able to tell their stories freely, some of them needed a lot of time and considerable prompting. The interview was completed when the participant had finished narrating his/her story and the researcher had no more topics to discuss. The interviews ranged in length from 30 to 45 min.

### Data analysis

Each recorded interview was transcribed verbatim by EN. All the interviews were translated into English. GM and EN verified the transcription by re-reading the transcribed data while listening to the recorded data. Data analysis was guided by the thematic narrative analysis by Riessman [13]. Firstly, transcribed data from in-depth interviews were read through multiple times by the first author to familiarize herself with the data and identified preliminary ideas. Thereafter, a line-by-line analysis of transcribed data was conducted to make meaning of the collected data. Colours were assigned to sentences in the transcripts to indicate potential patterns. EN and BCM identified and extracted phrases and/or direct participant quotations from the data. Codes were sorted into corresponding groups under potential thematic categories, which were frequently renamed when a more appropriate title emerged. Coauthors met multiple times to discuss and review the identified themes in order to reach an agreement. Three experienced qualitative researchers (KW, GM, and AM) scrutinized the themes to verify that they accurately reflected the information that had been collected. These researchers were not involved in data analysis.

### Trustworthiness of data

Four criteria for data trustworthiness proposed by Lincoln and Guba [21] were used. These include: credibility, dependability, confirmability, and transferability. In this study, peer debriefing and member checking were used to promote the credibility of the findings. Hamilton [22] asserts that peer debriefing helps to manage interviewer bias and to assist with conceptual clarity during data analysis and manuscript writing. Peer debriefing was done among the research team members after data collection and it was also a useful strategy during both data analysis and manuscript writing. Member checking was done by probing for clarity during the interviews. The research team members agreed that this was easier and more appropriate for the participants as opposed to member checking post interview for interpretive validation. Furthermore, trustworthiness was achieved through the inclusion of excerpts from participants’ verbatim narratives. Dependability was achieved through the detailed reporting of all processes within the study. These processes include; the research methods, the detailed collection of data and the analysis of the data. It is indicated that to ensure transferability, thick descriptive narratives must be provided for readers to judge the applicability of the findings in other settings and the verbatim quotes included in this paper also serve this purpose [21] Furthermore, to establish confirmabililty, the research team kept records of the raw data: field notes, transcripts, and a reflexive diary to enable readers to make an audit trail of all the decisions taken during the research process. All the decisions agreed upon during the peer debriefing among members were documented. Transferability was accomplished by providing sufficient detailed information about the methodology, participants, and their contexts so that the reader could judge how transferable the findings were to other settings.

## Study findings

There were ten (10) participants aged 26 to 56, including five males and five females. Three participants earned a living through subsistence farming, while others worked part-time or were retired; five respondents were married, two were divorcees, one was a widow, and the remaining had never married. The respondents’ educational levels were as follows: one attended secondary school, eight reached primary school, and one didn’t attend any formal education. The shortest period lived with diabetes managed on insulin therapy was two years and the longest was twenty years. The shortest distance diabetic patient reached the hospital was ten kilometers and the longest distance was seventy-one kilometers.

**Fig. 1:**
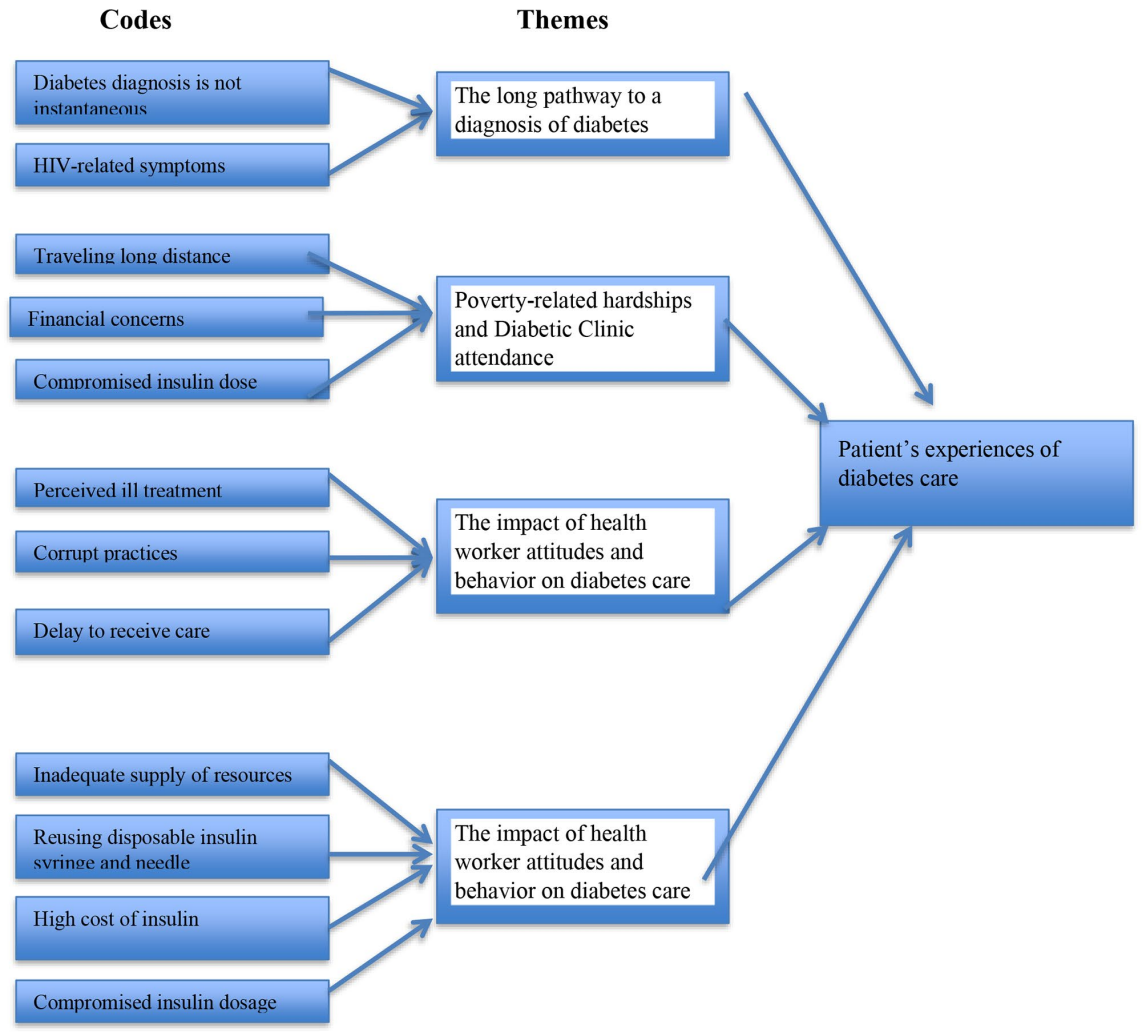
Coding tree

The analysis of the data yielded four themes: (1) the long pathway to a diagnosis of diabetes; (2) Poverty-related hardships and diabetic clinic attendance; (3) The impact of health worker attitudes and behavior on diabetes care; and (4) Low resources and their impact on self-management (see Fig. 1). The following is a description of the themes and text-based examples to illustrate them.

### The long pathway to a diagnosis of diabetes

The study findings reveal that participants suffered for a long time with symptoms of diabetes before the diagnosis was made. One characteristic feature was that some patients went to several hospitals before they were diagnosed. This is illustrated in the following interview excerpts:

*“I was very sick, I visited several hospitals (.); I was weak and lost weight”* (Participant #4-F-4-05).

*“I lost weight significantly and my feet were swollen (.) I visited different hospitals; I went to the Likuni Clinic and was told that I’m diabetic” (Participant #2-F-3-05)*.

The study findings reveal that the diagnosis of diabetes was not instantaneous. Initially, there was a clear weight loss that led family members, other people or even some of the patients themselves to suspect that they had HIV and AIDS, and the following voices illustrate this:

*“My family members thought that I had HIV/AIDS. I even doubted myself. I was weak, tired, lost weight, sweating and had shortness of breath”* (Participant #9-M-8-05).

Likewise, another participant had this to say:

*“Initially, I was just losing weight. I had lost so much weight and people used to say that I have contracted HIV”* (Participant #10-M-11-05).

Another participant mentioned that because he had HIV-related symptoms he was compelled to go for an HIV test.

“*So I was worried and had an HIV test; the result came out negative. I was surprised at what I have been suffering from. Then I was tested for sugar and was told that I have diabetes”* (Participant #10-M-11-05).

In this study, we found that HIV-related symptoms lead to stigmatization. Some of the participants reported that they sometimes found that other people in the community were openly gossiping about them.

*“Some individuals used to gossip that my husband was a womanizer and had infected me with AIDS (.) I was worried. This touched me so much”* (Participant #2-F-3-05).

Our study also found that patients begin to improve after they were commenced on insulin therapy. One of the participants expressed the following sentiments:

*“I do have hope as I have noticed that I’m getting better. I gained weight when I started using insulin after losing so much weight”* (Participant #9-M-8-05).

### Poverty-related hardships and diabetic clinic attendance

The study’s findings illustrate some of the difficulties that diabetics in rural areas face as a result of poverty. It is difficult for participants to visit the health facility on a diabetic clinic day as the excerpt illustrates:

*“Traveling a long distance to the hospital is difficult. When it’s a clinic day, my legs hurt due to traveling on foot the long distance*” (Participant #10-M-11-05).

*“You can’t believe that on a diabetic clinic day, I get to the hospital on foot due to lack of transport money, can you believe the distance from here to the central hospital? What else can I do? When it’s a clinic day, my foot hurts”* (Participant #9-M-8-05).

In this study, some of the patients compromised insulin dosages due to lack of funds to get more supply of insulin. as one of the participants gave the following account:

*“When I have remained with insufficient insulin and lack transport money to go to the hospital, I compromise the dosage of insulin by injecting myself reduced quantities”* (participant #3-M-4-05).

Another participant narrated that running out of medications led to negative health implications:

*“At times, medications would run out, then I would fall sick, as my sugar levels rose. I remember I fainted one day. When I got to the hospital, I was told that my sugar levels were very high. This is a result of having no transport money since I couldn’t go to the hospital to get more medications”* (Participant # 1-M-1-05).

### The impact of health worker attitudes and behavior on diabetes care

Numerous statements from the participants revealed that there is insufficient support at the diabetes clinic. They expressed their displeasure with the unfair treatment the rural-patients living with diabetes face unlike the rich patients and concluded that clinic day is a very stressful day. One participant said:

*“What is so painful is the tendency of the rich patients. They come from home and meet the doctor right away while those of us who get there earlier are still in the queue. We do nothing else other than just look at them (.) This is a great concern (.) we feel ill-treated at the Central hospital (.) So a clinic day is very stressful to the poor diabetic”* (Participant #5-F-5-05).

Most participants cited that health workers delay providing care. Some of the participants expressed the following sentiments:

*“A diabetic clinic day is a tough day for the diabetic. We usually arrive at the hospital in good time but the nurses and doctors take a long time to attend to us”* (Participant #6-M-6-05).

*“The other challenge is that we do not get the necessary support as patients. Ehh we are not treated well. The delay in running the clinic is unhealthy for the patients living with diabetes since we are food dependent” (Participant #4-F-4-05).*

One of the participants gave the following account as she shared the experience of a fellow patient living with diabetes who fainted while in the queue as a consequence of waiting for services while hungry, as indicated below:

*“It is a painful and stressful experience. It is not good for a a patient living with diabetes to go without food up to 11 am waiting to get tested yet the food is with us in the bag. You start feeling extreme hunger pangs and yet you have food in your bag. You experience low blood sugar level. I remember sometime back, a male patient waiting to be tested collapsed while in the queue due to low levels of blood sugar, as he had not taken breakfast”* (Participant #5-F-5-05).

Some of the participants reiterated that there are corrupt practices at the diabetic clinic, as indicated below:

*“There are some corrupt health care workers. They receive something from the patient; then they assist that particular patient right away”* (Participant #10-M-11-05).

*“When there is a corrupt doctor, we get back home at 3 pm yet we had arrived at the clinic at 6 am”* (Participant #5-F-5-05).

### Low resources and their impact on self-management

Although patients living with diabetes have access to the clinic, it was found that the inadequate supply of resources is a setback. For example, during the interviews participants explained that at the clinic, they are supplied with inadequate insulin syringes:

*“They give me 10 syringes; but it’s still not enough, because they finish quickly. I inject myself two times a day; 10 syringes last only 5 days”* (Participant #8-F-7-05).

*“Today, the hospital has run out of syringes; so I have been given medicine only and no syringes. I have to look for money so that I can buy syringes from the pharmacy (.)A set of 10 syringes is sold at MK1, 200.00. I get stressed if the syringes are unavailable at the hospital”* (Participant #3-M-4-05).

Some of the participants narrated that health workers instructed them to reuse a disposable insulin syringe and needle due to a lack of resources. One of the participants said:

*“Sometimes I’m given few syringes; then they (health workers) tell us to use one syringe for even five days”* (Participant #5-F-5-05).

Additionally, the participants themselves stated that they felt obliged to repeatedly reuse a disposable syringe until they felt pain because of the few syringes that were supplied.

*“I re-use one syringe until I feel pain when injecting; then I stop using it. This time I realized that it was worn out and I should use a new injection”* (Participant #2-F-3-05).

It is likely that sometimes patients may develop infections on injection sites due to repeated use of the syringes. One of the participants had this to say:

“*I was admitted to Kamuzu Central Hospital for a week because I had this wound on my thigh where I inject insulin. Ahhh, I couldn’t walk, I experienced pain, and the wound had pus on it”* (Participant #5-F-5-05).

The high cost of insulin was a major concern for the participants in this study, as the following excerpt illustrates:

*“If there is no medication, I get very worried because I cannot afford to buy insulin. The support I get from my relatives is inadequate, so can’t buy the medicine. Insulin is expensive in pharmacies, it’s not cheap; 1 bottle of Lente costs MK22, 000.00; I require two bottles per month; soluble insulin costs MK15 000.00 and I need 1 bottle, so the total is Mk59, 000; this treatment is for just one month”* (Participant #3-M-4-05).

*“The challenge we encounter at Kamuzu Central Hospital is the unavailability of medication. Therefore, we are asked to buy the medicine at the pharmacy yet we don’t have money (.) I get worried when there is no insulin at the hospital (.) I can’t afford to buy the medicine since don’t have such a sum of money”* (Participant #4-F-4-05).

The study findings reflect that some of the patients have no other choice but to miss insulin doses when their insulin runs out, which negatively impacts their health. One of the participants gave the following account:

*“I missed doses for up to 3 weeks due to a lack of insulin. I was admitted when I got to the hospital because my blood sugar was very high, I was sick ehhh. This was my bad experience”* (Participant #1-M-1-05).

Some of the participants would resort to improvising drinking a lot of water when insulin is not available, as the excerpt illustrates:

*“If there is no insulin, I just take more water and get to the hospital frequently to check the availability of drugs whilst I continue drinking a lot of fluids”* (Participant #3-M-4-05).

Some of the participants felt that the HIV/AIDS clinic received more attention and resources from the hospital. In contrast, they felt that diabetes clinic is often neglected. One of the participants expressed the following sentiments:

*“The other problem is that HIV clinics get too much attention; resources are always available to our friends who attend the clinic. In many cases, the diabetic clinic always runs short of medications and syringes. Sometimes, we are requested to buy insulin or syringes” (Participant#9-M-8-05)*.

## Discussion

This study explored the experiences of rural-based patients living with diabetes receiving care at a tertiary facility’s diabetic clinic in Malawi. Our study highlights the problems that rural-based patients living with diabetes encounter in accessing diabetes care. We found that participants are rarely presented with opportunities for the early diagnosis of diabetes. Therefore, the findings bring to light the existing deficiencies in diabetes diagnosis in resource-poor settings like Malawi. The delay in the diagnosis of diabetes may be attributed to an underdeveloped healthcare system with poor diagnostic facilities [ [23]. Consequently, patients suffer for a long time with symptoms such as weight loss and are suspected to have HIV and AIDS. The patients additionally suffer from the stigma and discrimination associated with HIV and AIDS. Malawi adopted the Essential Health Care Package (EHP) as an approach to health care delivery. An Essential Package of Health Services (EPHS) is defined as the package of services that the government is providing or is aspiring to provide to its citizens in an equitable manner [24]. The package outlines the government’s priority health interventions that patients need at every level of the healthcare delivery system [25]. It is a basic or minimum health care service [26]. However, the literature reveals that, as basic as it may appear, there are some implementation challenges. For example, in Malawi, there is evidence that the majority of the services are underprovided and underfunded covering on average 57% of the necessary costs [27] for diagnosis and illness management and access to dependable laboratory services. Access to dependable laboratory services and safe, suitable, and accurate diagnostics is crucial for diagnosis and illness management [28]. However, there is evidence that poor supply chain management of point-of-care (POC) diagnostics is one of the more frequent issues in some LMICs. Research evidence reveals complete stockouts of blood glucose tests among other diagnostics [29]. Results of national health facility surveys of pathology and laboratory medicine (PALM) and imaging diagnostics revealed a country-level availability of 31·6% for Malawi [30]. The absence of POC diagnostics in most healthcare facilities in Malawi hinders the prompt diagnosis of diabetes. Additionally, there is evidence that the proportion of health professionals who are knowledgeable about crucial diagnostic and treatment methods for EHP diseases is low [31].

In the present study, the average distance to the healthcare facility where the diabetic clinic took place was 35 km (km), and the longest distance was 70 km. Patients traveled long distances to access diabetes care, and clinic visits imposed financial burdens on them since diabetes care is offered at district and tertiary facilities. Therefore, decentralizing diabetes care to lower-level primary care facilities can improve access to diabetes care, especially in rural Malawi [32]. In addition, the distances we report in our study are longer than those reported in Assayed, Muula, and Nyirenda’s [9] study, where patients traveled almost 10 km to access the diabetes services in Blantyre. Our study further revealed that sometimes patients missed clinic visits due to a lack of transportation money. This sometimes led to insulin stockouts and sometimes patients reduced their insulin dosage to ensure that their medication lasted longer. This negatively affected their glycaemic control causing ill health and frequent hospitalization. These findings are supported by Oleyende [33] who revealed in a study that poor rural-based patients living with diabetes find it hard to physically get to a health resource setting; hence, patients tend to give up. They go to the health center only when they experience severe symptoms of the illness. Non-clinic attendance is associated with poor monitoring of patients and poor control of blood sugar [34]. No wonder patients in this study complained of feeling dizzy, losing consciousness because of poor glycaemic control. Oloyede [35] discovered that rural-based patients living with diabetes experience what has been referred to as ‘access stress’ where structural barriers; poor distribution of health services; distance and geography prevent them from successfully meeting the demands of diabetes self-management. For a chronic illness like diabetes, this may have a negative adverse effect on patients as it may lead to the rapid onset of diabetic-related complications.

We also found that participants encounter health workers who are unsympathetic. Patients living with diabetes perceive that they are unfairly treated and that they have to wait a long time while hungry to receive attention. This resulted in feelings of frustration with the public health care system. Metta et al. [36] also obtained similar findings in a study conducted in Tanzania, whereby patients living with diabetes were inconvenienced and had discomforts associated with having to wait for so long to access the services.

The study findings flag up the problem of corrupt practices in healthcare settings in Malawi. Even though the literature on health workers’ corrupt practices in diabetic clinics at public hospitals is rare; it does not mean that health workers do not practice the vice. The United Nations Development Programme [37] argues that in some countries, patients commonly make informal payments to health care professionals for better services, and the imposition of such a “fee” on “free” health care services has a negative impact on access to health services.

We observed that although patients living with diabetes have access to the diabetic clinic, the inadequate supply of resources for diabetes becomes a hindrance to effective self-management. Bui et al. [38] also revealed that tertiary level public hospitals in Malawi experience frequent stock-outs of oral medications and short-acting soluble and intermediate-acting Lente insulin. In this study, participants reported that insulin is costly and unaffordable in Malawian pharmacies. Similar challenges are reported in studies conducted in sub-Saharan Africa [39–41]. Like in a study done by Metta et al. [42]; this study identified that some of the participants compromised their insulin dosages, which can negatively impact on their health. In most low and middle income countries (LMICs), access to insulin remains a challenge, with availability and affordability being the two main obstacles [43]. In this study, participants felt that there were inequalities in the allocation of resources between the HIV/AIDS clinic and the diabetes clinic. These findings resonate with the assertion by Reid and Tsima [44]. Furthermore, evidence suggests that inequality has a profound impact on patients living with diabetes to the extent that some “wish they had HIV/AIDS” [45].

Additionally, our study found that participants repeatedly reused a disposable insulin syringe and needle due to insufficient resources. The literature identifies that repeated use of insulin syringes and needles by patients living with diabetes is a common practice particularly in resource-limited countries [46]. Mainly, unreliable and insufficient supplies and costs might lead to the syringes or needles being reused [47]. However, the World Health Organization [48] recommends the use of a new, single-use syringe and needle as it provides the highest level of safety to the recipients. In contrast, there is evidence that repeated reuse of insulin needles increases the risk of infection [49, 50]. It is not surprising that one of the participants in this study was hospitalized and managed for a wound infection at the injection site that could be attributed to infection from repeatedly reusing a contaminated syringe and needle. Our findings further expose that although insulin syringes are labeled single-use only; health workers in Malawi provide “unofficial” guidelines advising patients living with diabetes to reuse insulin syringes and needles due to a scarcity of resources. Ademe and Mekonnen [47] also reported similar findings in Ethiopia. Guterres et al. [51] recommend that health workers should provide adequate guidance on syringe and needle re-utilization if reusing insulin syringes and needles is unavoidable. Due to inadequate supplies of syringes, infection prevention should be emphasized at the Clinic by educating patients on hand washing.

### Strengths and limitations

This study is among the limited studies that have reported on the experiences of rural-based patients living with diabetes on diabetes care within low-and medium-income countries. The main limitation of the study is that it was conducted at one public tertiary healthcare facility, as such, the experience of diabetes patients might be different in other healthcare settings, and transferability of the findings to such settings may not be possible. There is a need to extend the study to all public tertiary healthcare settings in Malawi.

## Conclusions

We conclude that the study findings portray a high unmet need for diabetes care at a tertiary hospital. There is a need for the introduction of point-of-care testing to improve access to an early diabetes diagnosis. This approach has been shown to improve the significant health care gap in the diagnosis of diabetes in developing countries. Health workers should screen patients for diabetes in their general clinical assessments of patients presenting with persistent unspecific symptoms. Additionally, there is a need for the Ministry of Health to expand awareness campaigns among the population so that people are well informed about the signs and symptoms of diabetes for early diagnosis before diabetes symptoms and life-threatening complications set in. Diabetes care should be decentralized from tertiary facilities to lower-level primary care facilities to improve access to diabetes care for rural-based diabetic patients.

The study findings flag up gaps in the provision of diabetes services in Malawi’s government hospitals that negatively impacted on perceived well-being of rural-based patients living with diabetes. Therefore, further research is required in the following areas: (1) explore diabetes health services in Malawi’s government hospitals, which may isolate issues regarding the services rendered and do necessary improvements. (2) Perspectives of health care workers regarding diabetic care as this study did not involve health care providers.

### Electronic supplementary material

Below is the link to the electronic supplementary material.

Supplementary Material 1

## Data Availability

Data is available with the corresponding author and may be made available upon request.

## References

[1] Mathers CD, Loncar D (2006). Projections of global mortality and Burden of Disease from 2002 to 2030. PLoS Med.

[2] Wicks AC, Castle WM, Gelfand M. Effect of time on the prevalence of diabetes in the urban African of Rhodesia. Diabetes [Internet]. 1973 Oct [cited 2021 Mar 24];22(10). Available from: https://pubmed.ncbi.nlm.nih.gov/4743469/?dopt=Abstract. 10.2337/diab.22.10.733 4743469

[3] IDF. IDF Diabetes Atlas [Internet]. 2015 [cited 2019 Jul 24]. Available from: https://www.idf.org/e-library/epidemiology-research/diabetes-atlas/13-diabetes-atlas-seventh-edition.html.

[4] Msyamboza KP, Mvula CJ, Kathyola D (2014). Prevalence and correlates of diabetes mellitus in Malawi: population-based national NCD STEPS survey. BMC Endocr Disord.

[5] Malawi National Statistical Office, ICF International (2017). Malawi demographic and health survey 2015–2016.

[6] Prince AJ, Amberbr A, Musicha C, Crampin C, Kayuni-Chihana N, Mwansambo C. Prevalence of obesity, hypertension, and diabetes, and cascade of care in sub-saharan Africa: a cross-sectional, population-based study in rural and urban Malawi. The Lancet Diabetes & Endocrinology. 2018;6(3). 10.1016/S2213-8587(17)30432-1 PMC5835666 29371076

[7] George SR, Thomas SP (2010). Lived experience of diabetes among older, rural people. J Adv Nurs.

[8] Hornsten A, Lundman B, Selstam EK, Sandstrom H (2005). Patient satisfaction with diabetes care. J Adv Nurs.

[9] Assayed AA, Muula AS, Nyirenda MJ (2014). The quality of care of diabetic patients in rural Malawi: a case of Mangochi district. Malawi Med J.

[10] Creswell JW, Plano C, V (2011). L. Designing and conducting mixed methods research.

[11] Daiute C (2014). Narrative inquiry: a dynamic approach.

[12] Haydon G, Browne G, van der Riet P (2018). Narrative inquiry as a research methodology exploring person centred care in nursing. Collegian.

[13] Riessman CK. Narrative Analysis In: The Qualitative Researcher’s Companion [Internet]. 10.4135/9781412986274.

[14] Government of the Republic of Malawi. Health Sector Strategic Plan II 2017–2022: Towards Universal Health Coverage. 2017.

[15] Polit DF, Beck CT (2017). Nursing research: Generating and assessing evidence for nursing practice.

[16] Moule P, Goodman M (2014). Nursing research.

[17] Creswell JW (2013). Qualitative inquiry and research design: choosing among five approaches.

[18] LoBiondo-Wood G, Huber J (2014). Nursing research: methods and critical Appraisal for evidence-based practice.

[19] Janghorban R, Latifnejad Roudsari R, Taghipour A (2014). Pilot study in qualitative research: the Roles and values. J Hayat.

[20] Van Teijlingen E, Hundley V (2002). The importance of pilot studies. Nurs Standard.

[21] Lincoln YS, Guba EG (1985). Naturalistic inquiry.

[22] Hamilton JB (2020). Rigor in qualitative methods: an evaluation of Strategies among Underrepresented Rural Communities. Qual Health Res.

[23] Gill GV, Mbanya JC, Ramaiya KL, Tesfaye S (2008). A sub-saharan african perspective of diabetes. Diabetologia.

[24] Wright J (2015). Essential package of health services country snapshot: Malawi.

[25] WHO Africa. Ministry of Health Launched Basic Package for Essential Health Services [Internet]. 2010. Available from: https://www.afro.who.int/news/ministry-health-launched-basic-package-essential-health-services.

[26] Waddington C (2013). Essential health packages: what are they for? What do they change?.

[27] Bowie C, Mwase T (2011). Assessing the use of an essential health package in a sector wide approach in Malawi. Health Res Policy Syst.

[28] World Health Organization. Manual for procurement of diagnostics and related laboratory items and equipment [Internet]. 2013. Available from: https://apps.who.int/iris/handle/10665/255568.

[29] Kuupiel D, Tlou B, Bawontuo V, Drain PK, Mashamba-Thompson TP. Poor supply chain management and stock-outs of point-of-care diagnostic tests in Upper East Region’s primary healthcare clinics, Ghana. PLoS ONE. 2019;14(2). 10.1371/journal.pone.0211498 PMC6392218 30811407

[30] Yadav H, Shah D, Sayed S, Horton S, Schroeder LF (2021). Availability of essential diagnostics in ten low-income and middle-income countries: results from national health facility surveys. The Lancet Global Health.

[31] Mueller DH, Lungu D, Acharya A, Palmer N. Constraints to implementing the essential health package in Malawi. PLoS ONE. 2011;6(6). 10.1371/journal.pone.0020741 PMC3114780 21695115

[32] Makwero MT. Delivery of primary health care in Malawi. Afr J Prim Health Care Fam Med [Internet]. 2018 Jun 21 [cited 2020 Feb 4];10(1). Available from: https://www.ncbi.nlm.nih.gov/pmc/articles/PMC6018651/. 10.4102/phcfm.v10i1.1799 PMC6018651 29943590

[33] Oloyede O. The management of diabetes among the Rural Poor in South Africa. Afr Sociol Rev. 2013;17(2).

[34] Pastakia SD, Nuche-Berenguer B, Pekny CR, Njuguna B, O’Hara EG, Cheng SY (2018). Retrospective assessment of the quality of diabetes care in a rural diabetes clinic in western Kenya. BMC Endocr Disorders.

[35] Oloyede O (2013). The management of diabetes among the rural poor in South Africa. Afr Sociol Rev / Revue Africaine de Sociologie.

[36] Metta E, Bailey A, Kessy F, Geubbels E, Haisma H. Illness experiences of diabetes in the context of malaria in settings experiencing double burden of disease in southeastern Tanzania. PLoS One [Internet]. 2017 May 25 [cited 2020 Aug 14];12(5). Available from: https://www.ncbi.nlm.nih.gov/pmc/articles/PMC5444834/. 10.1371/journal.pone.0178394 PMC5444834 28542578

[37] United Nations Development Programme. Fighting corruption in the health sector – methods, tools and good practices [Internet]. 2013. Available from: http://www.undp.org/content/undp/en/home/librarypage/democratic-governance/anti-corruption/fighting_corruptioninthehealthsector/.

[38] Bui TD, Kadzakumanja O, Munthali C (2014). Mobilizing for the Lilongwe diabetes peer support Programme in Malawi. Malawi Med J.

[39] Metta E, Bailey A, Kessy F, Geubbels E, Hutter I, Haisma H (2015). In a situation of rescuing life: meanings given to diabetes symptoms and care-seeking practices among adults in Southeastern Tanzania: a qualitative inquiry. BMC Public Health.

[40] Mwangome MN, Geubbels E, Klatser P, Dieleman M (2016). I don’t have options but to persevere. Experiences and practices of care for HIV and diabetes in rural Tanzania: a qualitative study of patients and family caregivers. Int J Equity Health.

[41] Hjelm K, Mufunda E (2010). Zimbabwean diabetics’ beliefs about health and illness: an interview study. BMC Int Health Hum Rights.

[42] Metta E, Haisma H, Kessy F, Geubbels E, Hutter I, Bailey A (2015). It is the medicines that keep us alive: lived experiences of diabetes medication use and continuity among adults in Southeastern Tanzania. BMC Health Serv Res.

[43] Ewen M, Joosse HJ, Beran D, Laing R (2019). Insulin prices, availability and affordability in 13 low-income and middle-income countries. BMJ Global Health.

[44] Reid MJA, Tsima B (2014). It would have been better if I had HIV instead of diabetes. SAMJ: South African Medical Journal.

[45] Men C, Meessen B, van Pelt M, Damme WV, Lucas H (2012). I wish I had AIDS: a qualitative study on access to health care services for HIV/AIDS and diabetic patients in Cambodia. Health Cult Soc.

[46] Guterres CM, Rollin GAFS, Ribeiro RA, Bastos GN, Lima KM, Barrionuevo F (2015). Reuse of disposable syringes and needles in patients with type 2 diabetes. Diabetol Metab Syndr.

[47] Ademe M, Mekonnen Z (2014). Repeated reuse of insulin injection syringes and incidence of bacterial contamination among diabetic patients in Jimma University Specialized Hospital, Jimma, Ethiopia. Asian Pac J Trop Disease.

[48] World Health Organisation. Guideline on the use of safety-engineered syringes for intramuscular, intradermal and subcutaneous injections in health care setting. 2016. 27748094

[49] Bassetti S, Battegay M (2004). Staphylococcus aures infections in injection drug users: risk factors and prevention strategies. Infection.

[50] Nowakowska M, Jarosz-Chobot P, Polanska J, Machnica L (2007). Bacterial strains colonizing subcutaneous catheters of personal insulin pumps. Pol J Microbiol.

[51] Guterres CM, Rollin GA, Ribeiro RA, Kuhmmer R (2015). Reuse of disposable syringes and needles in patients with type 2 diabetes. Diabetol Metab Syndr.

