# Supplementary material for: Patient’s experiences of diabetes care at a tertiary health facility in Lilongwe, Malawi
Source: BMC Health Serv Res. 2023 Oct 12;23:1093. doi: 10.1186/s12913-023-10039-z (PMC10571416; doi:10.1186/s12913-023-10039-z)
Supplement: Supplementary file 1 — Supplementary Material 1 [file 12913_2023_10039_MOESM1_ESM.docx]

## Interview Guide

| **Study Title** |
| --- |

“Living with insulin-dependent diabetes: Narratives of rural-based adult Diabetic Patients , Lilongwe, Malawi”.

| **Researchers** |
| --- |

Ellen Nkambule, , [nkambule2019ellen@kcn.unima.mw](mailto:nkambule2019ellen@kcn.unima.mw), Kamuzu College of Nursing

Dr. Gladys Msiska, [gladysmsiska@kcn.unima.mw](mailto:gladysmsiska@kcn.unima.mw) ,Kamuzu College of Nursing

Dr . Annie Msosa, [anniemsosa@kcn.unima.mw](mailto:anniemsosa@kcn.unima.mw) ,Kamuzu College of Nursing.

**Contact details:** Kamuzu College of Nursing, Private bag 1

Lilongwe, Malawi.

Cell: 0888 395 377/ 099 850 2573/088 480 7721

| **Section A: Demographic data** |
| --- |

- 1. **Gender**

Male (1)

Female (2)

- 1. **Age**

18-29 (1)

30-39 (2)

40-49 (3)

50 years and above (4)

**3. Religion**

Roman Catholic (1)

C.C.A.P. (2)

Pentecostal (3)

Evangelical (4)

Islam (5)

SDA (6)

Others (7) Specify…………………………

**4. Marital status**

Single (1)

Married (2)

Divorced (3)

Widowed (4)

**5. Highest level of education**

None (1)

Primary (2)

Secondary (3)

Tertiary (4)

**6. Source of income**

Subsistence farming (1)

Business (2) Specify………………………………

Retired (3)

Others (4) Specify………………………………

**5. Duration of living with diabetes on insulin therapy**

>6months- 2years (1)

2-4years (2)

5-7years (3)

8-10years (4)

More than 10 years (5)

| **Section B: Grand tour questions** |
| --- |

**Note:** The grand tour questions below will guide the interviews and probing questions will be asked where necessary.

1. How would you explain diabetes mellitus in your own words?
2. The diabetes diagnosis, how did that make you feel?
3. How do you perceive your illness experience in general?
4. How do you perceive living with insulin-treated diabetes in rural areas?
5. What has your experience of living with insulin-treated diabetes in the rural areas been like from the time of diagnosis up to now?
6. What self-care management behaviours do you carry out at home?
7. What’s the hardest part of having your disease?
8. What kind of support do you get as you live with the disease in the rural areas?
9. From your experience can you share some practical issues that promote and hinder coping with insulin-dependent diabetes from rural setting?

**THANKS FOR YOUR PARTICIPATION!**
